# Preparation and evaluation of Vinpocetine self-emulsifying pH gradient release pellets

**DOI:** 10.1080/10717544.2017.1388453

**Published:** 2017-10-18

**Authors:** Mengqi Liu, Shiming Zhang, Shuxia Cui, Fen Chen, Lianqun Jia, Shu Wang, Xiumei Gai, Pingfei Li, Feifei Yang, Weisan Pan, Xinggang Yang

**Affiliations:** a Department of Traditional Chinese Medicine, Shenyang Pharmaceutical University, Shenyang, China;; b Department of Pharmacy, Shenyang Pharmaceutical University, Shenyang, China;; c Key Laboratory of Ministry of Education for TCM Viscera-State Theory and Applications, Liaoning University of Traditional Chinese Medicine, Shenyang, China

**Keywords:** Self-emulsifying drug delivery system, pH gradient pellet, oral bioavailability, poorly water-soluble drugs, Vinpocetine

## Abstract

The main objective of this study was to develop a pH gradient release pellet with self-emulsifying drug delivery system (SEDDS), which could not only improve the oral bioavailability of Vinpocetine (VIN), a poor soluble drug, but reduce the fluctuation of plasma concentration. First, the liquid VIN SEDDS formulation was prepared. Then the self-emulsifying pH gradient release pellets were prepared by extrusion spheronization technique, and formulation consisted by the liquid SEDDS, absorbent (colloidal silicon dioxide), penetration enhancer (sodium chloride), microcrystalline cellulose, ethyl alcohol, and three coating materials (HPMC, Eudragit L30D55, Eudragit FS30D) were eventually selected. Three kinds of coated pellets were mixed in capsules with the mass ratio of 1:1:1. The release curves of capsules were investigated *in vitro* under the simulated gastrointestinal conditions. In addition, the oral bioavailability and pharmacokinetics of VIN self-emulsifying pH gradient release pellets, commercial tablets and liquid VIN SEDDS were evaluated in Beagle dogs. The oral bioavailability of self-emulsifying pH gradient release pellets was about 149.8% of commercial VIN tablets, and it was about 86% of liquid VIN SEDDS, but there were no significant difference between liquid SEDDS and self-emulsifying pH gradient release pellets. In conclusion, the self-emulsifying pH gradient release pellets could significantly enhance the absorption of VIN and effectively achieve a pH gradient release. And the self-emulsifying pH gradient release pellet was a promising method to improve bioavailability of insoluble drugs.

## Introduction

Oral drug delivery has been the most extensive and convenient administration route for treatment of many diseases (Agrawal et al., 2014). However, in recent years, nearly 40% of the new drug compounds show a poor solubility in water, which brings about low oral bioavailability (Gursoy & Benita, 2004; Cui et al., 2009). Therefore, enhancing the water solubility of drugs is the key to the new drug development to improve its oral bioavailability. In order to overcome these difficulties, lots of strategies were presented, including the use of cosolvents, permeation enhancers and reducing the particle size; formulations as nanoparticles, liposomes, micronization, inclusion complex, solid dispersions, etc. (Aungst, 1993; Abdalla & Mader, 2007; Curatolo et al., 2009; Wang et al., 2010; Morgen et al., 2017).

In recent years, more and more attention has been gathered in a lipid formulation, self-emulsifying drug delivery system (SEDDS), it can effectively improve the water solubility and bioavailability of insoluble drugs (Kommuru et al., 2001; Wang et al., 2010; Qi et al., 2014; Dokania & Joshi, 2015). SEDDS is an isotropic mixture of oil, surfactant, cosurfactant, and drugs that forming fine oil-in-water emulsion under the gentle agitation which is similar to human gastrointestinal tract (Kang et al., 2004; Grove et al., 2006; Wang et al., 2010). After oral administration, small droplets formed by SEDDS can increase the interfacial surface area with the drug in the gastrointestinal tract, thereby enhancing the absorption and oral bioavailability of drugs (Cui et al., 2009; Wang et al., 2010; Wei et al., 2012; Qi et al., 2014). Nevertheless, there are still some shortcomings of the SEDDS, such as low stability, drug leakage and precipitation, not easy to store, etc. Solidifying of SEDDS, like self-emulsifying tablet and pellets, is to combine the advantages of solid dosage forms with liquid SEDDS, including the improvement of stabilization and bioavailability, which possesses a good development prospects (Nazzal et al., 2002; Newton et al., 2007; Wang et al., 2010; Qi et al., 2014). However, SEDDS solidification has problems with burst release, so SEDDS could be prepared into sustained release pellets or gradient release pellets. For the past few years, pH-dependent drug delivery system has been more popular in formulation research field, usually prepared many dosage forms for specific purpose, like sustained-release drug delivery system and some targeted delivery systems (Yang et al., 2004a). The self-emulsifying coated pellets we prepared were put in a capsule to obtain a pH gradient release drug delivery system. These pH-sensitive polymers dissolved at different pH value in the gastrointestinal tract, therefore the drug could be released in different part of the gut. Furthermore, the treatment process would be extended which depends on the dissolution characteristics of the material, so as to achieve the desired therapeutic effect (Yang et al., 2004b).

Vinpocetine (VIN) is a derivative of alkaloid apovincamine, separated from vinca leaves. VIN is claimed to increase cerebral-blood flow in local cerebral ischemia area, antiplatelet aggregation and ability to neuroprotective (Bereczki & Fekete, 1999; Ribeiro et al., 2003; Jincai et al., 2014). Therefore, it usually has been employed as a clinic drug for the treatment of cerebrovascular and cerebral degenerative diseases (Luo et al., 2006; Patyar et al., 2011). At the same time, however, it has a short elimination half-life (1-2 h), the greater first-pass effect (75% metabolism in liver) and the existing formulation is typically a tablet containing 5 mg of VIN with a poorly oral bioavailability (about 6.7%), thus all of these extremely limited the clinic development of VIN (Ribeiro et al., 2003; Ding et al., 2015). As a consequence, it would be far-reaching significance to find a suitable dosage form to improve the water solubility and oral bioavailability of VIN for the treatment of cerebrovascular and cerebral degenerative diseases.

Thus, the aim of current study was to develop a pH gradient release formulation about self-emulsifying pellets containing VIN. The dosage form could not only improve the water solubility and oral bioavailability of VIN, but also reduce the fluctuations of plasma concentration and prolong the drug-treating time. We use three different coating materials to dissolve the pellets at different pH conditions and test the release of the drug *in vitro*. Furthermore, the bioavailability of VIN in self-emulsifying pellets was evaluated in comparison with commercial VIN tablets and liquid SEDDS in the fasted Beagle dogs.

## 2. Material & method

### 2.1 Material

VIN was obtained from Harbin Sanlian Pharmaceutical Factory (Heilongjiang, China). Dulbecco’s Modified Eagle’s medium and Nonessential amino acids (DMEM) were purchased from GIBCO (USA). Solutol HS 15 was supplied by BASF (Ludwigshafen, Germany). Transcutol P was obtioned from Castris (Gattefosse, France). Ethyl oleate was purchased from Beijing Changcheng Chemical Ltd. (Beijing, China). Fetal bovine serum (FBS) was gained from Hycolone (USA). Penicillin, streptomycin, trysin and ethylenediaminetertraacetic acid (EDTA) were purchased from Sigma (USA). Transwell^®^ nunclone™ was supplied by COSTAR (Camgridge, MA, USA). Eudragit L30D55 and Eudragit FS30D were obtioned from Evonik Degussa. HPMC was gained from Colorcon. Sucrose, mannitol and sodium bicarbonate?were purchased from Bodi chemical co, LTD (Tianjin, China). Other materials were Solutol HS 15 (BASF, Ludwigshafen, Germany), Transcutol P (Castris, Gattefosse, France), ethyl oleate (Beijing Changcheng Chemical Ltd., Beijing, China), fetal bovine serum (FBS) (Hyclone, USA), penicillin, streptomycin, trypsin, ethylenediaminetetraacetic acid (EDTA) (Sigma, USA), Transwell^®^ nunclon™(COSTAR, Cambridge, MA, USA), Eudragit L30D55 and Eudragit FS30D (Evonik Degussa), HPMC (Colorcon), sucrose, mannitol, sodium bicarbonate (Bodi chemical co., LTD, Tianjin, China), All other reagents were of analytical grade.

### 2.2 Method

#### 2.2.1 Preparation of liquid SEDDS

Based on a series of experimental studies, the optimized liquid SEDDS formulation was selected: Solutol HS15 50%, Transcutol P 35%, Ethyl Oleate 15% and VIN 20% (Cui et al., 2009). These materials were prepared as SEDDS according to the following steps:

- The Solutol HS15, Transcutol P and Ethyl Oleate were put together in a glass vessel, and then stirred until obtain a homogeneous mixture.
- The VIN was added into the abovementioned mixture and was stirred at room temperature until a desired solution was got.
- The prepared liquid SEDDS was stored at indoor temperature for standby.

#### 2.2.2 Cells culture

The Caco-2 cells were cultured in 75cm^2^ flasks by DMEM with high glucose at 37 °C (Pichardo et al., 2017). Supplement with 10% FBS, 1% nonessential amino acids, 1% penicillin streptomycin, and 5% CO_2_ (relative humidity at 90%). For the transport experiments of VIN, cells were seeded on a Millicell^TM^ membrane at 5 × 10^4^/cm^2^. The culture medium were replaced every 2 days for the first week after seeding, and then replaced every day, until Day 21. The cells were suitable for the experiments only when the transepithelial electrical resistance (TEER) values were >600Ω/cm^2^ and 3H mannitol apparent permeability coefficient (Papp) < 1 × 10^−6 ^cm/s. That indicated the Caco-2 cell monolayers were intact and not leaked (Sha et al., 2005; Feng & Betti, 2017).

#### 2.2.3 Transport assays

The transportation of VIN across the Caco-2 cells was investigated by employing the monolayers which were 21 days post seeding. Before the experiment, removed the medium from Transwell**^®^** and the monolayers were washed twice with HBSS at 37 °C. For the transmembrane experiment of VIN solution, 0.5 ml of the VIN solution was added on the apical (AP) side and 0.5 ml of blank HBSS without VIN was added on the basolateral (BL) side. For the transmembrane experiment of liquid VIN self-microemulsifying drug delivery systems (SMEDDS), the method as above mentioned, 0.5 ml of liquid VIN SMEDDS was added on AP side and 0.5 ml of blank HBSS was added on the BL side. The VIN solution were freshly prepared by dissolved in dimethyl sulfoxide DMSO and the liquid VIN SMEDDS were prepared as mentioned in 2.2.1. Put the Transwell**^®^** plate in a shaker at 37 °C and shaken at 50 rpm (Sha & Fang, 2004). During the transport test, samples were taken 200 μl from the BL side, respectively, in different time (10 min, 20 min, 30 min, 45 min, 1 h, 1.5 h, 2 h, 3 h, 4 h, 5 h) and then adding an equal volume of fresh HBSS solution in the BL side immediately as a replacement. The drug concentration was determined by high performance liquid chromatography (HPLC) and each test was tripled.

#### 2.2.4 Preparation of self-emulsifying pH gradient release pellets

Different kinds of excipients (absorbent, diluents, adhesives, etc.) were screened through the single factor investigation, and then self-emulsifying pellets were prepared with the following steps:

- The MCC (diluents) was added into the abovementioned SEDDS liquid to give it complete absorption and then mixed well with the remaining excipients in equal incremental method.
- 40% EtOH was added to prepare the damp mass.
- The damp mass was extruded at a velocity of 30 rpm and spheronizing of 800 rpm at 5 min, after 40 °C drying, screening the pellets over 20-30 mesh sieve.

Then three kinds of coating materials were selected (HPMC, Eudragit L30D-55, Eudragit FS 30 D) to prepare self-emulsifying pH gradient release pellets by fluidized bed coating method. These coating excipients could dissolute at different pH condition to achieve the effect of pH gradient release. Preparation of self-emulsifying pH gradient release pellets mainly following the processes:Firstly, three coating excipients were prepared as a coating solution. And then, 10 g pellets which were mentioned above were placed in a fluidized bed, and adjusted the jet pressure to 0.5 MPa. Finally, appropriate amount of coating solution was applied, respectively, the pellets were coated at 35 °C at a constant current pump as 1.0 ml/min.

#### 2.2.5 Evaluation of self-emulsifying pH gradient release pellets

##### 2.2.5.1 Particle size of pellets and emulsions

The particle size and distribution of the pellets and the emulsions prepared with the optimum preparation conditions were investigated. The 0.5 g self-emulsifying pellet samples of three batches were placed in beakers, respectively, diluted with 100 ml 0.1 mol/L HCl, after fully emulsified, the 0.8 μm microporous membrane was used for filtration.

##### 2.2.5.2 ζ-Potential

Particle surface charge is an important factor which affecting the emulsion stability. In this study, the **ζ**-potential analyzer was used to determine the **ζ-**potential of three batches self-emulsifying pH gradient release pellet samples.

##### 2.2.5.3 Emulsification rate

The self-emulsification rate was determined by turbidity method. As the following two steps:

- Three batches of self-made pellet samples were taken 0.5 g, respectively, added to 200ml 0.1mol/L HCl (Chinese Pharmacopoeia 2015 Part IV. Third method device).
- Respectively, at each time point 2, 5, 10, 20, 30, 45, 60 min, 5ml liquid was sampled, after filtering by 0.8 μm microporous membrane, the filtrate was collected and the absorbance was measured at 550 nm.

##### 2.2.5.4 Differential scanning calorimetry (DSC) analysis

Differential scanning calorimetry (DSC) was performed to measure the physical status of VIN in the self-emulsifying pH gradient release pellets, to determine whether the pellets made after solidification of the liquid SEDDS are still present in an amorphous state.

The VIN self-emulsifying pellets and blank self-emulsifying pellets (no drugs) were prepared. A part of blank pellets were crushed and the VIN was dispersed in them to obtain a physical mixture. The VIN, the blank pellets, the physical mixture, and the self-emulsifying pH gradient release pellets were placed on an appropriate amount in crucibles of aluminum and then sealed, programed temperature 10 °C/min and temperature range of 30-300 °C (Doshi & Shrivastava, 1993).

##### 2.2.5.5 Scanning electron microscope

The morphology of the self-emulsifying pH gradient release pellets were analyzed by Scanning electron microscope(SEM) to observe the appearance of the coated pellets (Krupa et al., 2014). The micrographs were taken at 50 × and 60 × magnification.

#### 2.2.6 *In vitro* release study

The dissolution test was carried out by paddle method stated in the Chinese Pharmacopoeia 2015. First, appropriate amount of the three kinds of coated pellets were weighed by weight ratio of 1: 1: 1 into the hard capsule. The release study of the self-emulsifying pH gradient release pellets were carried on in the simulated human gastrointestinal pH condition (hydrochloric acid solution, pH1.2, 2 h; phosphate buffer salt solution, pH6.8, 3 h; phosphate buffer salt solution, pH7.4, 3 h) at 100 rpm and the temperature of the dissolution medium was constant at 37 ± 0.5 °C. Next, sampling at different time (0,1,2,3,4,5,6,7,8 h), in the meantime, the same amount of dissolution medium was supplying. The samples were filtered through a micropore membrane (0.8 μm) and the filtrate was collected and analyzed by ultraviolet (UV) spectrophotometer to examine the release drug concentration (Wang et al., 2010). The test was performed six times.

#### 2.2.7 *In vivo* bioavailability study

In this study, the three-cycle 3 × 3 cross-test method was used to investigate the bioavailability of self-emulsifying pH gradient release pellets. Six Beagle dogs (supplied by Shenyang Pharmaceutical University Animals Center, weighing 10–12 kg) were randomly divided into three groups, and fasting 12 h prior but free drinking water before the experiment. The three groups were oral-administrated with commercial tablets, liquid SEDDS and self-emulsifying pH gradient release pellets, respectively, and the three administrations all contained 15 mg VIN, as shown in Table 1, and in a crossover design with 2 weeks washout between dosing. No other drugs could be taken during the 2 weeks before the experiment and the experiment period for this study, to prevent interference with the experimental results.

Blood samples were collected approximate 5 ml from the foreleg veil of Beagle dogs and placed in a centrifugal tube with heparin at 0, 0.25, 0.5, 0.75, 1, 1.5, 2, 2.5, 3, 4, 5, 6, 8, 10, 12, and 24 h after dosing. The collected blood samples were then immediately centrifuged at 4000 rpm for 10 min. The separated plasma was stored at −20° C until analysis. The concentration of VIN in plasma was determined by HPLC.

#### 2.2.8 Statistics

The data were demonstrated with mean ± SD (standard deviation) and the analysis was performed using Student’s two-sided *t*-test. Significant differences were considered at *p* < 0.05.

## 3. Result & discussion

### 3.1 Drug delivery of Caco-2 monolayers

**Figure 1.**
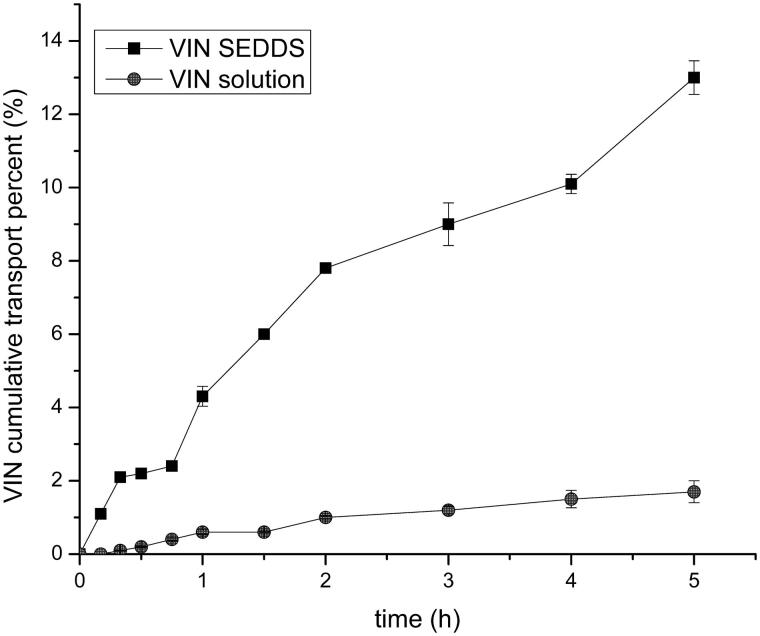
Transepithelial cumulative percent across Caco-2 monolayers at 37 °C.

The Caco-2 cell line is a type of human cloned colonic adenocarcinoma cell and it is regularly used as a model for evaluating drug transmembrane transport. The VIN solution used as a control group was compared with the efficiency of VIN SEDDS for Caco-2 cell transmembrane transport test. The test results are shown in Figure 1. During the study period, the transmembrane transport rate of VIN solution was no more than 2%, while the transport rate of liquid VIN SEDDS increased distinctly with time and was obviously higher than that of VIN solution. That revealed the SEDDS could significantly enhance the transport of the drug (*p* < .05). Furthermore, the *Papp* of the liquid VIN SEDDS was about 254 times of the VIN solution. These result demonstrated the SEDDS could possibly improve the transport of drugs in the body, thereby increasing the bioavailability. Therefore, for the study of the insoluble drug VIN, the SEDDS formulation may greatly improve its pharmacological activity that may offer an effective help with treatment of cerebrovascular disease and improve the cerebral circulation.

### 3.2 Evaluation the properties of self-emulsifying pH gradient release pellets

#### 3.2.1 Characterization of emulsions

**Figure 2.**
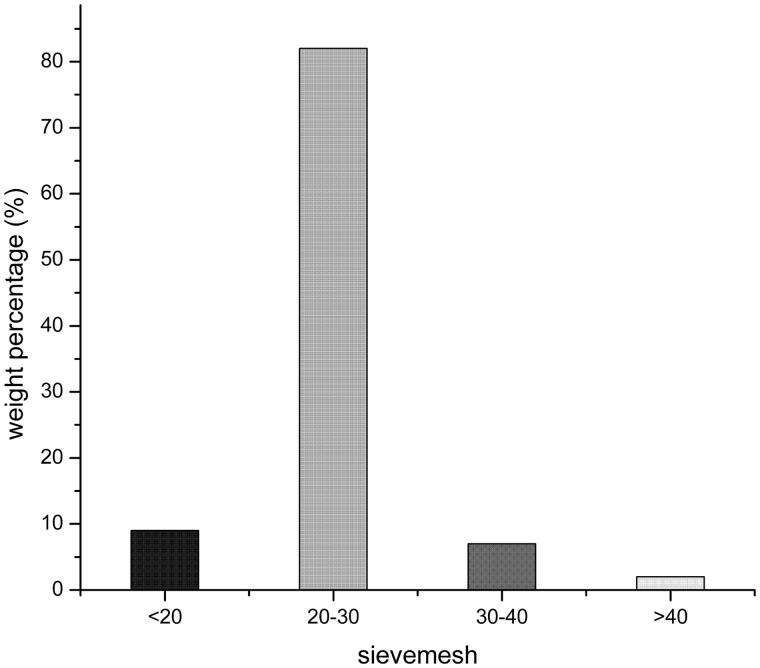
Particle size distribution.

In this experiment, the result about particle size distribution of self-emulsifying pellets was shown in Figure 2. The experiment pellets were screened for 20-30 mesh sieves. In addition, the average particle size of the emulsion which formed after emulsification of three batches pellet samples were measured, the average particle size was 317.1 nm. Particle surface charge is an important influence factor for the stability of the emulsion. The zeta potentials of the three batches of pellet samples were −2.04, −1.87, and −1.66 mv, respectively, with an average of −1.85 mv.

#### 3.2.2 DSC analysis

**Figure 3.**
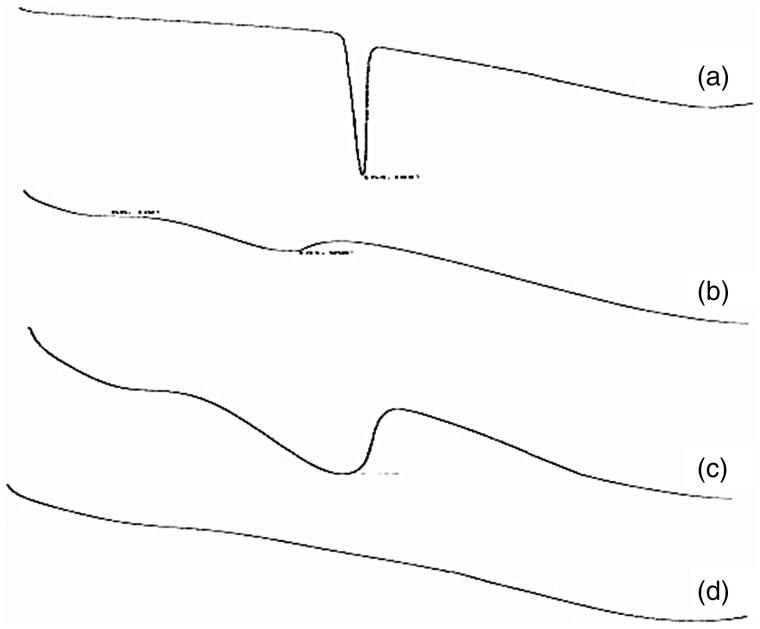
DSC thermograms of (a) VIN (b) blank self-emulsifying pellets (c) physical mixture (d) VIN self-emulsifying pH gradient release pellets.

The physical state of the four substances (VIN, blank self-emulsifying pellets, physical mixture, and VIN self-emulsifying pH gradient release pellets) was analyzed by DSC. Figure 3 shows the consequence. As shown, the endothermic peak of the physical mixture was the superposition of VIN and the endothermic peak of the blank excipient, indicating that VIN was existence in the physical mixture only in the crystalline state. While in VIN self-emulsifying pellets preparation, the disappearance of the VIN endothermic peak demonstrates the drug existing as amorphous state. As a conclusion, amorphous form can enhance the dissolution of poorly soluble drugs and improve the bioavailability, it may be one of the reasons that VIN self-emulsifying pH gradient release pellets has better effect about enhancing bioavailability and water solubility (Chen et al., 2016; Miriyala et al., 2017).

#### 3.2.3 Scanning electron microscope

**Figure 4.**
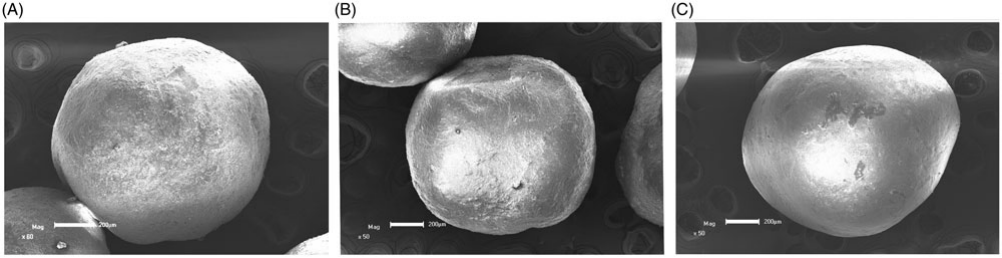
Scanning electron micrographs of pellets (A) pellet coated with HPMC (B) pellet coated with Eudragit L30D-55 (C) pellet coated with Eudragit FS30D.

The results of SEM were shown in Figure 4. From picture (A), (B), and (C), the pellets were coated with HPMC, Eudragit L30D-55, and Eudragit FS30D, respectively, they were similar in appearance and no significant difference. The pellets were protected from being released at specific pH conditions to achieve the prospective gradient release.

### 3.3 *In vitro* release study

**Figure 5.**
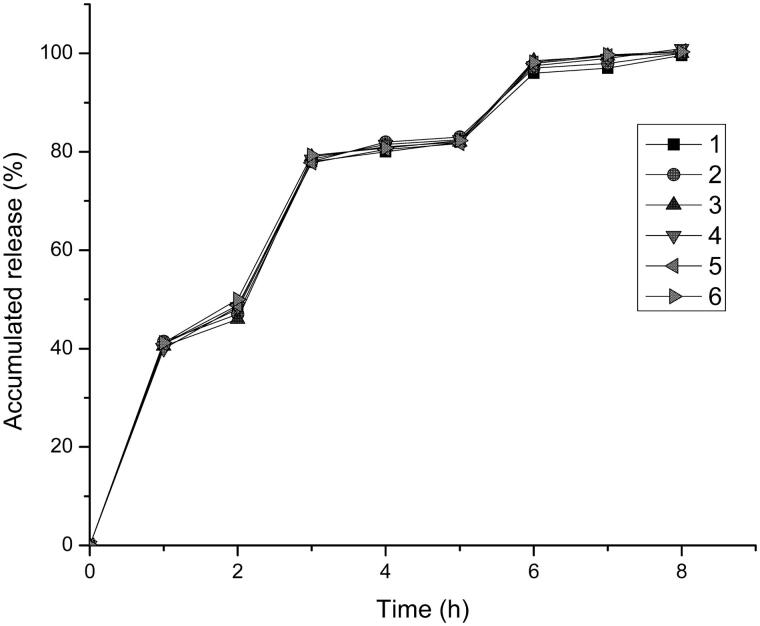
The release curve in the simulated gastrointestinal pH conditions (*n* = 6).

Figure 5 shown the release of the drug in simulated gastrointestinal conditions (pH1.2; pH6.8; pH7.4) during the study period. HPMC as a water-soluble material, the solubility also well in the stomach pH value (pH 1.2) (Cole et al., 2001; Aho et al., 2017). HPMC could be quickly dissolved in the stomach, so that the drug could be released rapidly from the pellets to achieve the efficacy. The two enteric polymers were almost insoluble in 0.1 mol/L HCl (pH 1.2) (Patra et al., 2017), indicated that the two polymers would not be dissolved in the stomach. Another coating material we used was Eudragit L30D-55, there was a good release at intestine pH value (pH 6.8) (Hu et al., 2006; Rujivipat & Bodmeier, 2012), it could be rapidly dissolved in the intestine without affecting the drug release. The last kind of coating material we chose for the formulation was Eudragit FS 30 D. The threshold pH of it was about 7, the polymer against the dissolution at gastrointestinal environmental which pH was less than 7 and dissolved in distal of intestine the pH was over 7 (Ji et al., 2007). The results indicated that self-emulsifying pellets possessing the capacity of pH gradient release, and the release rate is good. It is clear to see that, after about 6 h, the drug has already completely released.

### 3.4 *In vivo* bioavailability test

**Figure 6.**
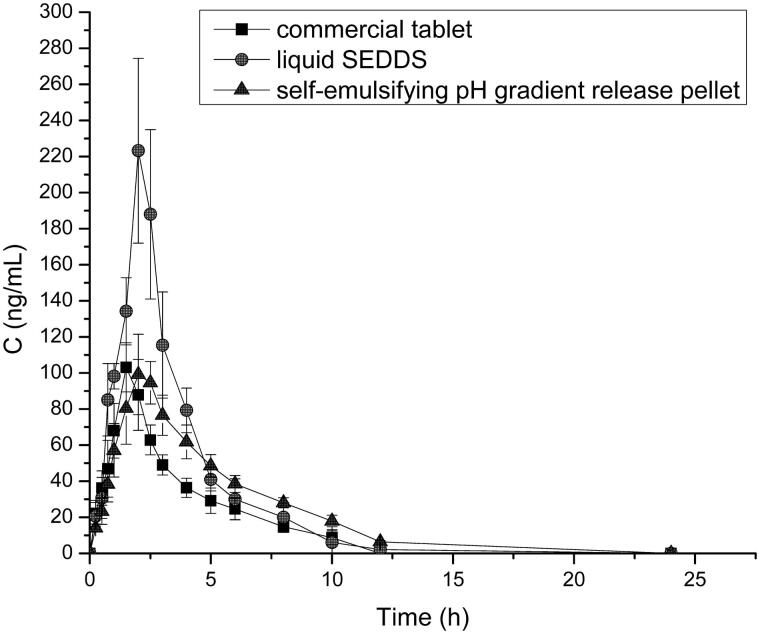
Profiles of mean plasma concentration-time after oral administration of VIN commercial tablet, liquid SEDDS, and self-emulsifying pH gradient release pellet (*n* = 6).

Compared with commercial tablets and liquid SEDDS preparations, the pharmacokinetic study on SEDDS pellets were carried out in six healthy Beagle dogs. The mean plasma concentration versus time curves of VIN in commercial tablets, liquid VIN SEDDS and self-emulsifying pH gradient release pellets and the main pharmacokinetic parameters were shown in Figure 6 and Table 2, respectively (Cui et al., 2009). The results demonstrate the T_max_ of the two SEDDS preparations was all longer than that of the commercial tablets, but after reaching T_max_, only the self-emulsifying pellets showed a sustained release effect, indicating that self-emulsifying pellets can make the drug lasting effect longer in the body. And the C_max_ of the liquid SEDDS was 2.2-fold higher than that in self-emulsifying pellets and commercial tablets. What’s more, the plasma concentration fluctuations of liquid SEDDS were very large, which was harmful to some special diseases, such as cardiovascular and cerebrovascular diseases. In addition, the AUC_0–t_ of liquid SEDDS and self-emulsifying pellets were 1.8- and 1.4-fold higher than that of the commercial tablets. The relative bioavailability of liquid SEDDS and self-emulsifying pellets of VIN compared with commercial VIN tablets were (174.2 ± 17.3)% and (149.8 ± 23.4)%, respectively. Both of SEDDS formulations can significantly improve the oral bioavailability of VIN, and the bioavailability of self-emulsifying pellets was slightly lower than that of liquid SEDDS, but there was no significant difference between them, and it was probably because the pellets in the body to release drugs need to disintegrate, moreover, there may be liquid SEDDS *in vivo* absorption can partially avoid the first effect of liver and so on.

**Table 1.** Studies schedule in Beagle dogs for different formulations.

	Dog number					
Period	1	2	3	4	5	6
1	A	A	B	B	R	R
2	B	R	R	A	A	B
3	R	B	A	R	B	A

A: liquid SEDDS; B: self-emulsifying pH gradient release pellet; R: commercial tablet.

**Table 2.** Pharmacokinetic parameters of commercial tablet, liquid SEDDS and self-emulsifying pellets after oral administered.

Parameters	Commercial tablet	Liquid SEDDS	Self-emulsifying pellet
T_max_ (h)	1.667 ± 0.258	2.167 ± 0.258*	2.083 ± 0.376*
C_max_ (ng/mL)	110.340 ± 8.715	242.296 ± 41.700*	110.614 ± 10.385
AUC_0–t_ (ng/mL·h)	349.155 ± 37.813	630.942 ± 41.616*	476.937 ± 20.374*

* *p* < .05 compared with commercial tablets.

The self-emulsifying pH gradient release pellets not only improve the oral bioavailability, but also reduce the fluctuation of plasma concentration and prolonging the time of drug action. There is a good effect on the treatment of chronic diseases such as cerebrovascular and brain degeneration. Moreover, the self-emulsifying pH gradient release pellets combining the advantages of SEDDS and solid dosage forms, it might be make the SEDDS technology a vaster development and application.

## Conclusion

In this study, first, the liquid VIN SEDDS was prepared, then the self-emulsifying pH gradient release pellets was prepared with three kinds of coating materials, HPMC, Eudragit L30D-55 and Eudragit FS 30 D, three coated pellets were placed into the capsule at a ratio of 1: 1: 1. *In vitro* dissolution test, the result showed that the self-emulsifying pellets could achieve the desired effect about pH gradient release and the drug could achieve a good release effect. And after 6 h the release of drugs almost to completely release in the simulation of the human gastrointestinal. In *in vivo* tests, a comparison of the AUC_0–t_ between self-emulsifying pellets and commercial tablets indicated that the bioavailability of VIN self-emulsifying pH gradient release pellets were about 149.8% of commercial tablets. In this study, the use of SEDDS technology not only enhances the drug solubility and bioavailability, but also good for the treatment of cerebrovascular or other diseases. The self-emulsifying pH gradient release pellets provide a potential use value for drugs with the large fluctuations in plasma concentration, just like VIN.
